# Proteome Profiling of Cerebrospinal Fluid and Machine Learning Reveal Protein Classifiers of Two Forms of Alzheimer’s Disease Characterized by Increased or Not Altered Levels of Tau

**DOI:** 10.1016/j.mcpro.2025.101025

**Published:** 2025-06-30

**Authors:** Elisabetta Scalia, Matteo Calligaris, Margot Lo Pinto, Salvatore Castelbuono, Matilda Iemmolo, Vincenzina Lo Re, Giulia Bivona, Tommaso Piccoli, Giulio Ghersi, Simone Dario Scilabra

**Affiliations:** 1 Proteomics Group of Ri.MED Foundation, Research Department IRCCS ISMETT (Istituto Mediterraneo per i Trapianti e Terapie ad Alta Specializzazione), Palermo, Italy; 2 Department of Biological, Chemical and Pharmaceutical Sciences and Technologies (STEBICEF), University of Palermo, Palermo, Italy; 3 Department of Medical Area (DAME), Università degli studi di Udine, Udine, Italy; 4 Research Department, IRCCS ISMETT (Istituto Mediterraneo per i Trapianti e Terapie ad alta Specializzazione), Palermo, Italy; 5 Department of Engineering, University of Palermo, Palermo, Italy; 6 Neurology Service, Department of Diagnostic and Therapeutic Services, IRCCS ISMETT (Istituto Mediterraneo per i Trapianti e Terapie ad Alta Specializzazione), University of Pittsburgh Medical Center (UPMC), Palermo, Italy; 7 Department of Biomedicine, Neurosciences and Advanced Diagnostics, University of Palermo, Palermo, Italy

**Keywords:** Alzheimer’s disease, cerebrospinal fluid, data-independent acquisition (DIA) proteomics, machine learning, protein classifiers

## Abstract

Alzheimer’s disease (AD) is a multifactorial neurodegenerative disorder that presents with heterogeneous clinical and pathological features, necessitating improved biomarkers for accurate diagnosis and patient stratification. In this study, we applied a data-independent acquisition-based proteomics workflow to cerebrospinal fluid (CSF) samples from 138 individuals, including AD patients with high (Aβ+/tau+) or normal (Aβ+/tau−) CSF tau levels, and non-AD controls. Analysis using an Astral mass spectrometer enabled unprecedented proteome depth, identifying 2661 proteins with high data completeness. Comparative proteomic profiling revealed distinct protein signatures for Aβ+/tau+ and Aβ+/tau− subtypes. These findings were validated using an independent internal cohort and further corroborated with publicly available datasets from larger external AD cohorts, demonstrating the robustness and reproducibility of our results. Using machine learning, we identified a panel of 15 protein classifiers that accurately distinguished the two AD subtypes and controls across datasets. Notably, several of these proteins were elevated in the preclinical stage, underscoring their potential utility for early diagnosis and stratification. Together, our results demonstrate the power of data-independent acquisition proteomics on the Astral platform, combined with machine learning, to uncover subtype-specific biomarkers of AD and support the development of personalized diagnostic strategies.

## Highlights

• Astral MS and DIA enabled deep and comprehensive CSF proteome profiling.
• Distinct CSF proteomes identified in Aβ+/tau+ and Aβ+/tau− AD subtypes.
• Machine learning revealed a 15-protein panel distinguishing AD subtypes.
• Classifier differentiates Aβ+/tau+ and Aβ+/tau− from non-AD controls.
• The 15-protein signature holds promise as a biomarker panel for AD.

## Graphical Abstract

**Figure:**
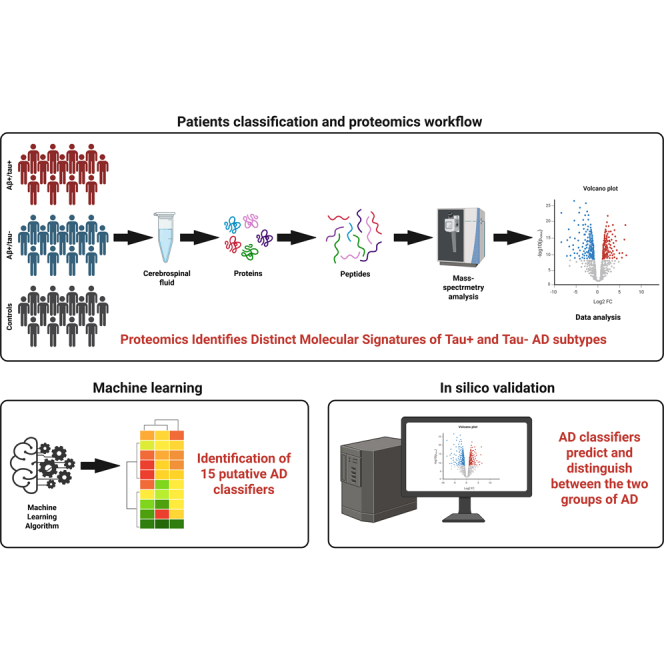

## In Brief

Deep DIA-based proteomics using high-resolution Astral MS enabled comprehensive profiling of cerebrospinal fluid from Alzheimer’s patients. Machine learning identified a 15-protein signature that distinguishes Aβ+/tau+ and Aβ+/tau− subtypes from each other and from non-AD controls. These findings highlight the potential of CSF proteomics to refine Alzheimer’s disease stratification and support precision medicine approaches.

Alzheimer’s disease (AD) is the most common form of dementia, a neurodegenerative disorder that inevitably leads to memory loss, cognitive decline, and ultimately death. AD is a true silent epidemic with devastating socioeconomic impacts. AD pathology is characterized by the cerebral accumulation of amyloid plaques and neurofibrillary tangles, the two hallmarks of the disease, which ultimately lead to neuronal cell death, shrinkage of brain tissue, progressive cognitive, and behavioral impairment (1). However, AD is now widely recognized as a multifactorial disorder, with multiple factors and biological pathways implicated beyond the classical amyloid cascade (2). This complexity highlights the urgent need for personalized medicine, as different subgroups of AD patients exist and treatments effective for one subgroup of AD patients may be ineffective to others. The identification of reliable biomarkers that enable accurate diagnosis, effective patient stratification, and better clinical trial design remains a key research priority (3, 4).

Cerebrospinal fluid (CSF), which reflects the biochemical environment of the brain, has become a valuable biofluid for investigating AD pathogenesis and patient stratification. In clinical practice, CSF is already used for the amyloid-tau-neurodegeneration (ATN) classification system of AD patients, which assesses levels of amyloid-β (A) and phosphorylated tau (T), along with the evaluation of neurodegeneration (N) by brain imaging, to biologically define AD and distinguish it from other dementias. In recent years, CSF has also been extensively investigated to better elucidate the molecular mechanisms of AD, identify novel biomarkers and characterize distinct AD subtypes (5). Multiple analytical platforms have been developed to profile the CSF proteome for these purposes, ranging from targeted approaches like aptamer-based assays (*e.g.*, SomaScan) and proximity extension assays (*e.g.*, Olink) to unbiased methods like mass spectrometry-based proteomics. For instance, Olink-based proteomics, although limited to the quantification of 665 targeted proteins, has successfully uncovered CSF signatures that distinguish AD from mild cognitive impairment (MCI) as well as from other dementias, including dementia with Lewy bodies and frontotemporal dementia (6, 7, 8). Moreover, another study showed that this technique enabled the quantification of 1472 CSF proteins in individuals with either sporadic or genetic forms of AD, leading to the identification of distinct proteomic signatures associated with both forms of the disease (9).

Unlike targeted approaches, mass spectrometry-based proteomics offers a more comprehensive and unbiased analysis of the CSF proteome (10). A common issue in body fluid proteomics is the abundance of high-concentration proteins, which can hinder the detection of low-abundance biomarkers. Recent technological advancements, including improvements in mass spectrometer resolution, enhanced data acquisition, and more efficient processing techniques, have helped overcome longstanding challenges associated with analyzing complex protein samples with large dynamic ranges, such as CSF (11, 12). Thus, while earlier studies were limited to identifying only a few hundred proteins in biological fluids like CSF, modern proteomic workflows now enable greater proteome coverage and more extensive protein identification (10). Tandem mass tag labeling proteomics, combined with off-line high-pH reversed-phase fractionation and data-dependent acquisition (DDA), has allowed a deeper characterization of AD CSF, leading to the identification and quantification of thousands of proteins, and to the discovery of several distinct subtypes of the disease (13, 14). More recently, a proteomic workflow based on data-independent acquisition (DIA) for label-free quantification (LFQ) of cerebrospinal fluid (CSF) proteins has proven highly effective for in-depth CSF proteome profiling. Unlike earlier approaches, this method achieves high proteome coverage and depth without the need for extensive sample fractionation or depletion of abundant proteins (15, 16, 17). Notably, this method has enabled the identification of robust proteomic signatures of AD patients, which have been successfully validated across independent cohorts.

In this study, we applied the advanced DIA-based proteomic workflow not only to canonical AD patients, defined by decreased amyloid beta 1 to 42 (Aβ_42_) and increased total tau in the CSF, but also to a distinct subgroup of AD patients, who were cognitively indistinguishable and had similarly reduced Aβ_42_ levels, but showed normal CSF tau levels (Aβ+/tau+ and Aβ+/tau- AD patients). In addition, we analyzed CSF samples with an Astral mass spectrometer, which enabled unprecedented proteome depth and coverage. The CSF proteomic alterations observed in both Aβ+/tau+ and Aβ+/tau- AD patients using this workflow were highly consistent with findings generated by alternative proteomic workflows and mass spectrometry platforms. Furthermore, when combined with machine learning, this approach enabled the identification of a 15-protein CSF signature capable of distinguishing not only between AD patients and non-AD controls, but also between the two AD subtypes. This signature was confirmed in an internal cohort and further validated in silico using two independent, larger AD cohorts, all analyzed with distinct proteomic workflows and mass spectrometry platforms.

## Experimental Procedures

### Experimental Design and Statistical Rationale

For the proteomic profiling of CSF using liquid chromatography–tandem mass spectrometry (LC-MS/MS), samples were collected from 88 AD patients, consisting of 60 with high CSF tau levels (Aβ+/tau+, n = 61) and 27 with normal CSF tau levels (Aβ+/tau-, n = 28), along with 50 non-AD controls. In total, 138 samples were analyzed. The samples underwent tryptic digestion through filter-aided sample preparation (FASP), were desalting by stop-and-go extraction (STAGE) tipping, and then were resuspended in 0.1% formic acid (18, 19). Peptides from each sample were separated by HPLC, ionized through electrospray ionization and analyzed using a Vanquish Neo coupled online with an Astral mass spectrometer. Protein identification and LFQ were performed using DIA, and raw spectra were processed with the DIA-NN software. Statistical significance of protein alterations was assessed using Student’s *t* test for three pairwise comparisons: the Aβ+/tau + group versus non-AD controls; the Aβ+/tau-group versus non-AD controls; and the Aβ+/tau + group versus the Aβ+/tau-group.

To identify proteins significantly altered across the three groups—and thus potentially capable of distinguishing Aβ+/tau+ from Aβ+/tau-AD subtypes—we performed a multisample test ANOVA using Perseus software with default settings, which include a permutation-based false discovery rate (FDR) correction. This analysis identified 342 differentially abundant proteins, which were subsequently used to train a machine learning model to predict AD and differentiate between the Aβ+/tau+ and Aβ+/tau-subtypes. Feature selection process using a gradient-boosting algorithm (XGBoost) reduced this protein panel to 15 key classifiers. This signature was retrospectively validated for its ability to distinguish Aβ+/tau+, Aβ+/tau- and non-AD controls in a second cohort of patients (Aβ+/tau+, n = 14; Aβ+/tau-, n = 10; non-AD controls, n = 18). Samples from the validation cohort underwent FASP, HPLC separation, and DIA analysis; however, unlike the discovery cohort, they were analyzed using an Exploris 480 mass spectrometer. The resulting datasets (from the larger discovery cohort analyzed with the Astral platform and the smaller validation cohort analyzed with the Exploris 480) were further evaluated in silico using external datasets from the proteomic analysis of a larger AD cohort (13, 20).

### Selection and Classification of Patients

AD patients and control individuals were recruited from the Clinic for Cognitive Decline, Dementia, and Parkinsonism at the University Hospital Paolo Giaccone in Palermo, Italy, as previously described (21). All participants underwent a comprehensive assessment, including neurological and cognitive examination; fludeoxyglucose-18 - positron emission tomography; brain magnetic resonance imaging and a lumbar puncture for CSF collection. Inclusion criteria for the AD group were based on a diagnosis of MCI due to AD (22) or probable AD dementia with evidence of AD pathophysiological process (23). The 88 AD patients from the first cohort, and the 24 patients from the second, were further classified into Aβ+/tau+ and Aβ+/tau-groups according to the ATN classification (24). The Aβ+/tau + group showed reduced levels of Aβ42 and Aβ _40_/Aβ_42_ in the CSF (below the Aβ42 < 650 pg/ml and Aβ40/Aβ42 < 0.069 cutoff, respectively), along with increased total tau and phospho-tau (above t-tau >416 pg/ml and p-tau >61 ng/ml cutoff, respectively). Conversely, the Aβ+/tau-group had low levels of Aβ42 and Aβ40/Aβ42 in the CSF, t-tau, and p-tau levels similar to non-AD controls. No age difference was noted between the two groups. The control group consisted of 60 individuals in the first cohort and 18 in the second cohort without signs of AD, but with other neurological conditions, metabolic diseases, or substance abuse (21). The characteristics of the Aβ+/tau+, Aβ+/tau- and non-AD groups are summarized in Table 1. All participants provided written informed consent, and the study was conducted in accordance with the Declaration of Helsinki. The study protocol was approved by the Institutional Ethics Committee of the University Hospital "Paolo Giaccone Palermo" under the reference number Institutional Ethic Committee Palermo1 No. 07/2017.

**Table 1:** Demographic and biochemical characteristics of the study cohort

Cohort characteristics	Aβ+/tau + [n = 61]	Aβ+/tau- [n = 27]	Non-AD [n = 50]
Number of patients	61	27	50
Age, mean	70.5 ± 6	72.9 ± 6.5	65.6 ± 10
Aβ 1–42 (pg/ml), mean	456.6 ± 130.6	424.9 ± 103	1156 ± 397
Total tau (pg/ml). mean	785.5 ± 290.8	341.1 ± 72.7	329.7 ± 161.4
p-tau (pg/ml), mean	112.9 ± 52.58	48.39 ± 14.09	34.6 ± 14.5

The characteristics of the study cohort by age, CSF levels of Aβ40, Aβ42, total tau and phospho-tau.

### CSF Preparation for High-Resolution Proteomics

After collection, CSF samples were centrifuged at 400g for 20 min and subsequently stored at −80 °C until analysis. Samples were subjected to FASP using Vivacon Spin 10 kDa (Sartorius) (19). Briefly, 10 μg of proteins were reduced by the addition of 20 mM DTT (Thermo Fischer Scientific) in 100 mM Tris–HCl, 8 M urea pH 8.5 for 30 min at 37 °C. The proteins were then alkylated with 50 mM iodoacetamide (Thermo Fischer Scientific) for 5 min at room temperature and washed twice in 100 mM Tris–HCl, 8 M urea pH 8.0 at 14,000*g* for 30 min. Next, proteins were digested overnight with 0.2 μg LysC (Promega) in 25 mM Tris–HCl, 2 M urea pH 8.0, followed by digestion with 0.1 μg trypsin (Promega) in 50 mM ammonium bicarbonate for 4 h. The resulting peptides were desalted by stop-and-go extraction-tips with reverse phase C18 (Supelco Analytical Products, part of Sigma-Aldrich), and eluted in 40 μl of 60% acetonitrile (ACN) in 0.1% formic acid (18). The volume was then reduced in a SpeedVac (Thermo Fisher Scientific), and the peptides were resuspended in 20 μl of 0.1% formic acid.

### LC-MS/MS and Data Analysis

Two different setups were used for LC-MS/MS analysis. The first comprised a nanoLC system (Vanquish Neo UHPLC) coupled online to an Astral mass spectrometer, using a 75 μm × 150 mm DNV PEPMap Neo (instruments and column from Thermo Fischer Scientific), and the second comprised a nanoLC system (Vanquish Neo UHPLC) coupled online to an Exploris 480 mass spectrometer, using a 25 cm × 75 μm Acclaim PEPMap C18 column (instruments and column from Thermo Fischer Scientific). Peptide concentration was determined using a NanoDrop microvolume spectrophotometer (Thermo Fischer Scientific) before LC-MS/MS analysis.

#### Astral

Subsequently, 350 ng of peptides were separated using a 19 min binary gradient of water and ACN, both containing 0.1% formic acid, at variable flow rate (μl/min): (i) 0 to 6% ACN in 0.5 min at 650 μl/min; (ii) 6 to 8% ACN in 0.5 min at 600 μl/min; (iii) 8 to 11% ACN in 0.7 min at 520 μl/min; 11 to 21% ACN in 9.7 min at 520 μl/min; (iv) 21 to 36% ACN in 4.8 min at 520 μl/min; (v) 36 to 55% ACN in 0.5 min at 520 μl/min; (vi) 55 to 95% ACN in 2.3 min at 650 μl/min. DIA was performed using an MS1 full scan (380 m/z to 980 m/z) followed by 200 sequential DIA windows with a 3 m/z without overlap. Full scans were acquired at a resolution of 240000, with an automatic gain control (AGC) of 5 × 10^6^, and a maximum injection time of 5 ms. Afterward, 200 isolation windows were scanned at the fixed resolution of Astral detector 80000, with an AGC of 5 × 10^4^ and the maximum injection time set at 5 ms. Scan range (m/z) was set at 100 to 1000 and higher energy collision dissociation collision energy set at 25%.

Data analysis was performed using DIA-NN software (version 1.8.1) with a predicted library generated from an in silico digested human UniProt reference database (UP000005640_9606), downloaded on 24.07.2023. Digestion allowed cleavage at K and R, with up to two missed cleavages and a minimum peptide length of six. The maximum number of variable modifications was set to 1. N-terminal methionine excision and cysteine carbamidomethylation were enabled as fixed modifications, while methionine oxidation and N-terminal acetylation were enabled as variable modifications. Mass accuracy and MS1 accuracy were both set to 0.0 for automatic inference. Precursor charge range was set at 2 to 5. The precursor m/z range was set to 380 to 980, and the fragment ion m/z range to 100 to 1000. This library consisted of 20921 protein isoforms, 30958 protein groups, 6036186 precursors in 2850551 elution groups. The FDR for peptide and protein identification was set at 0.01%.

#### Exploris 480

One microgram of peptides was separated using a 132 min binary gradient of water and ACN, both containing 0.1% formic acid, at flow rate 0.250 μl/min: (i) 3 to 6% ACN in 3 min; (ii) 6 to 30% ACN in 90 min; (iii) 30 to 44% ACN in 20 min; (iv) 44 to 75% ACN in 20 min; (v) 75 to 95% ACN in 10 min at 0.500 μl/min. DIA was performed using an MS1 full scan (400 m/z to 1000 m/z) followed by 60 sequential DIA windows with a 1 m/z overlap and the window placement optimization option enabled. Full scans were acquired at a resolution of 120,000, with an AGC of 3 × 10^6^, and a maximum injection time of 50 ms. Afterward, 60 isolation windows were scanned at a resolution of 30,000, with an AGC of 8 × 10^5^, and the maximum injection time was set to “auto” for optimal cycle time. Collision induced dissociation fragmentation was achieved with 30% normalized HCD collision energy. Data analysis was performed using DIA-NN software (version 1.8.1) with a predicted library generated from an in silico digested human UniProt reference database (UP000005640_9606), downloaded on 24.07.2023. Digestion allowed cleavage at K and R, with up to two missed cleavages and a minimum peptide length of six. The maximum number of variable modifications was set to 1. N-terminal methionine excision and cysteine carbamidomethylation were enabled as fixed modifications, while methionine oxidation and N-terminal acetylation were enabled as variable modifications. Mass accuracy and MS1 accuracy were both set to 0.0 for automatic inference. Precursor charge range was set at 2 to 5. The precursor m/z range was set to 400 to 1000, and the fragment ion m/z range to 200 to 1800. This library consisted of 20921 protein isoforms, 30958 protein groups, 6036186 precursors in 2850551 elution groups. The FDR for peptide and protein identification was set at 0.01%.

### Data Analysis

LFQ was used for protein quantification and analyzed with Perseus (version 1.6.15.0–(25)). LFQ values were log2 transformed, and a two-sided Student’s *t* test was used to evaluate statistical changes in relative protein abundance between groups, with a significance threshold set at a *p*-value of less than 0.05. Protein groups were retained for statistical analysis only if they were quantified in at least 70% of the samples in one or more of the study groups. Gene ontology (GO) enrichment analysis of significantly altered proteins was conducted using the Enrichr and SynGO web tools (26, 27).

### Machine Learning for the Identification of Protein Classifiers of tau+ and tau-Subtypes of AD Patients

To identify proteins significantly altered across the three different groups (Aβ+/tau+, Aβ+/tau-, and non-AD controls), the mean of log2-transformed protein intensities of the Aβ+/tau+, Aβ+/tau-groups and non-AD controls were compared using multisample ANOVA with permutation-based FDR correction (q < 0.05, S0 = 0, randomization = 250). Applied to the Astral-generated dataset of 138 samples, this analysis revealed 342 proteins with significantly altered abundance in either AD subgroup compared to controls. These proteins were then standardized using the mean and standard deviation of the control group and expressed as z-scores, where 0 represents the control group mean, z > 0 indicates increased abundance and z < 0 indicates reduced abundance. Missing intensity values were imputed with zero. To identify the most informative proteins for AD classification, four supervised machine learning algorithms were evaluated: XGBoost (XGBoost python package v1.7.8.1), Random Forest, support vector machine (SVM), and k-nearest neighbors (KNN) (sklearn python library - v1.6.1) (28). The total dataset was randomly split into a training set (70% of the data) and a test set (30%). The ability of the classifiers in predicting the different forms of AD was evaluated on the test set using a confusion matrix and a receiver operating characteristic curve to visualize prediction results. XGBoost yielded the highest classification accuracy for differentiating Aβ+/tau+, Aβ+/tau-, and non-AD groups. It also enabled feature ranking via Shapley additive explanations (SHAP) values, identifying 15 proteins that give a contribution to the classification outcomes. These selected features were then applied to an independent, previously published proteomic dataset obtained from the AD Workbench—a publicly available resource from the Alzheimer’s Disease Data Initiative (ADDI)—comprising patient samples collected at the Alzheimer Center Amsterdam (ACA). Patient classification was conducted using the same four machine learning algorithms described above.

## Results

### Set up of a Proteomic Workflow for CSF Profiling

**Fig. 1:**
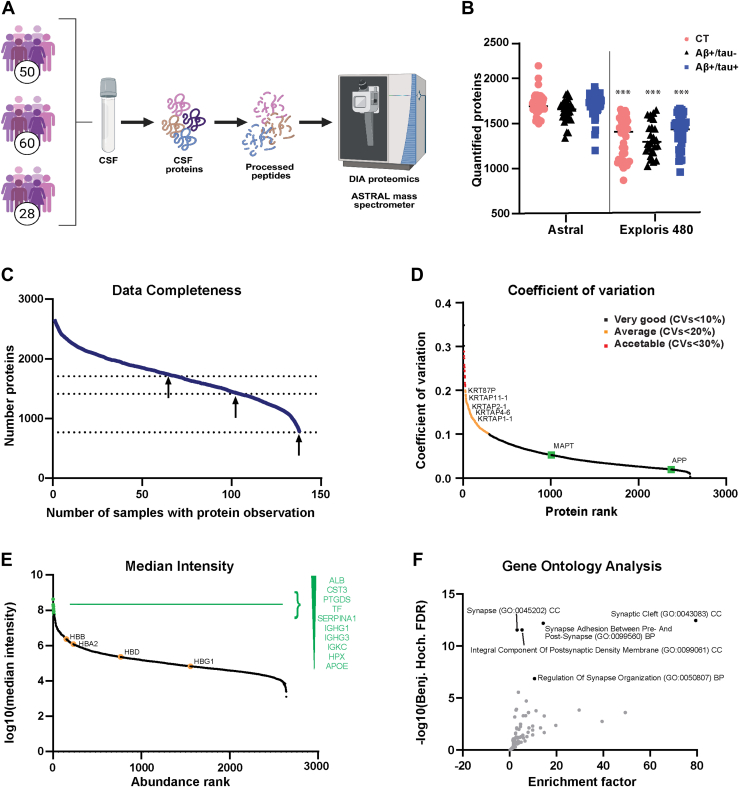
**Validation of the proteomic pipeline for CSF proteome profiling**. *A*, overview of the study groups and schematic proteomic workflow. The CSF of two subgroups of AD patients (Aβ+/tau + AD and Aβ+/tau-) and non-AD controls was analyzed. The total number of patients per group is depicted. *B*, number of proteins identified and quantified in Aβ/tau+ and Aβ/tau- AD patients, and non-AD controls (CT), by the Astral or the Exploris 480 platform. Statistical differences in the number of proteins quantified per group between platforms were assessed using a Student’s *t* test, comparing each group analyzed on Astral to the corresponding group analyzed on Exploris 480. (∗∗∗*p* < 0.005). *C*, data completeness curve. The number of proteins in the dataset (*Y* axis) depending on the minimum number of samples in which the proteins have each been quantified (*X* axis) is plotted. The *arrows* indicate 50%, 75%, and 100% data completeness. *D*, graph depicting the coefficient of variation (CV) for identified proteins, indicating the relative variability in protein expression levels across samples. Higher CV values reflect greater variability, while lower values suggest more consistent protein expression. Plasma contaminants were the most variable proteins (CV < 20–30%); APP and MAPT have a lower variability among samples (CV<5%). *E*, median CSF protein abundance distribution as calculated from MS intensities of quantified peptides of each protein. The top 10 most abundant proteins are named in *green*, and hemoglobins highlighted within the curve. *F*, gene ontology enrichment analysis of CSF proteins. Plot showing the -log10 of false discovery rates (FDRs) versus the enrichment factors. CSF, cerebrospinal fluid; AD, Alzheimer’s disease.

**Supplementary Figure 1:**
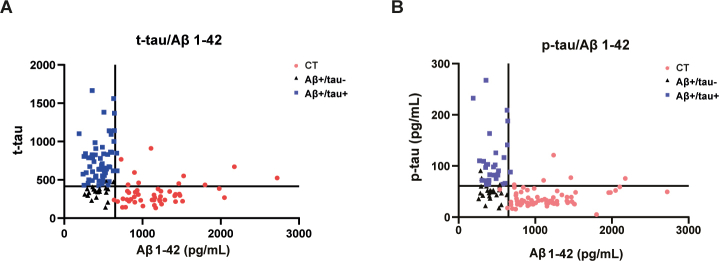
*A* and *B* CSF levels of Aβ42 were plotted against levels of phosphorylated tau (p-tau – *A*) or total tau (t-tau – *B*) for each individual involved in the study. This clearly separated three groups, Aβ+/tau+ AD patients with reduced Aβ42 and increased p-tau and t-tau, Aβ+/tau- AD patients with reduced Aβ42 and normal p-tau and t-tau, and non-AD with normal CSF levels of Aβ42 and p-tau (please note, a few patients clinically diagnosed as non-AD based on the NINCDS-ADRDA criteria exhibit CSF p-tau and t-tau levels above the threshold, likely as a consequence of other neurological conditions).

In this study, we profiled the CSF proteome of AD patients and non-AD controls using a recently developed, streamlined workflow that includes DIA and LFQ (17) (Fig. 1*A*). The cohort included 88 AD patients diagnosed according to the NINCDS-ADRDA criteria (24, 29) and 50 non-AD controls. AD patients were further stratified into two subgroups based on CSF levels of total and phosphorylated tau. Both subgroups exhibited decreased levels of Aβ_42_ compared to non-AD controls; one subgroup had elevated levels of tau and phosphorylated tau (Aβ+/tau+, n = 60), while the other showed normal levels (Aβ+/tau-, n = 28 patients) (supplemental Fig. S1). For each of the 138 samples, 10 μg of CSF protein (equivalent to ∼ 5–15 μl of CSF) was subjected to tryptic digestion via FASP, followed by DIA-based label-free quantification. Unlike the reference study by Bader *et al*. (17), which used DIA and was conducted using a Q-Exactive HF Orbitrap mass spectrometer, we used an Astral mass spectrometer, which is expected to provide enhanced performance by combining a quadrupole with a novel asymmetric track lossless analyzer. This design introduces an additional dimension of resolution by separating ions based on their time-of-flight. Using this platform, we identified and quantified a total of 2661 proteins across all groups, with an average of 1671 proteins per CSF sample. Specifically, we quantified an average of 1703 proteins in the Aβ+/tau + group, 1614 in the Aβ+/tau- and 1694 in the non-AD control group (Fig. 1*B*, supplemental Tables S1, and S2). DIA analysis showed high data completeness: 725 proteins were quantified in 100% of the samples, 1413 proteins in 75% of the samples, and 1708 proteins in 50% of the samples (Fig. 1*C*). These results indicate that, using a comparable sample preparation and acquisition workflow, the Astral mass spectrometer enabled deeper and more complete proteomic profiling of the CSF compared to the dataset of the reference study by Bader *et al*., which reported an average of 1233 quantified proteins across 197 CSF samples, with 100% completeness for 385 proteins, 75% completeness for 1050 proteins and 50% completeness for 1288 proteins (17).

**Supplementary Figure 2:**
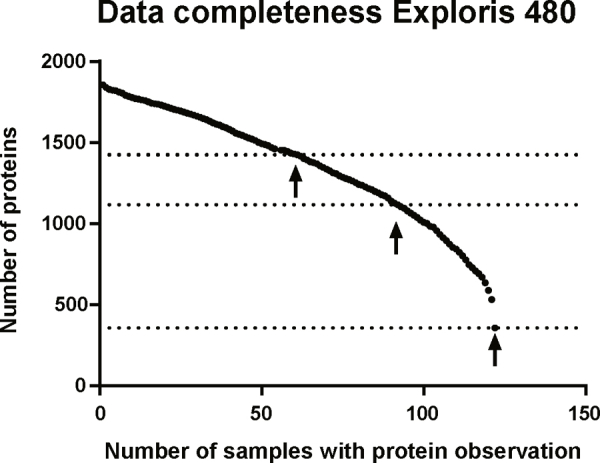
A Data completeness curve of CSF samples analysed by an Exploris 480 mass spectrometer. The number of proteins in the dataset (Y axis) depending on the minimum number of samples in which the proteins have each been quantified (X axis) is plotted. The arrows indicate 50%, 75%, and 100% data completeness.

To further validate the enhanced performance of the Astral-based workflow, we reanalyzed 122 of the 138 samples (the remaining samples were unavailable due to limited material) using an Exploris 480 mass spectrometer. This analysis identified 1855 proteins across all groups, with an average of 1353 proteins per sample (Fig. 1*B*). In terms of completeness, 355 proteins were detected in 100% of samples, 1115 proteins in 75%, and 1424 proteins in 50% (supplemental Fig. S2). Given the higher proteome coverage and data completeness achieved with the Astral mass spectrometer, we selected the Astral-derived dataset for all subsequent analyses.

To ensure the accuracy of CSF sample collection and their suitability for proteomic analysis, we evaluated total protein yield and intersample variability, while also monitoring for potential contaminants such as plasma-derived proteins and keratins. Plasma contaminants, including keratins (e.g. KRTAP2-2, KRTAP4-6, KRT34, and so on) exhibited higher variability, with a coefficient of variation (CV) between 10% and 20% (Fig. 1*D*). On the other hand, proteins relevant to AD, such as APP and MAPT (gene name of tau protein), showed low variability among the samples (coefficient of variation below 5%). This indicates that, despite the inherent variability in the sampling procedure—which can lead to variable levels of plasma contaminants that are difficult to completely avoid—the quality of the CSF samples was suitable for proteome profiling. The intensity of quantified proteins spanned six orders of magnitude, with the top 10 most abundant proteins (identified by gene name: ALB, CST3, PTGDS, TF, SERPINA1, IGHG1, IGHG3, IGKC, HPX, and APOE) contributing to 63.5% of the total protein intensity across all 2661 proteins in the dataset (Fig. 1*E*). To further confirm that the quantified proteins were functionally related to the brain, we conducted a GO analysis. The analysis revealed 114 common categories among identified proteins, with the most represented GOs pertaining to the “synaptic cleft” (GO:0043083) and “synapse adhesion between presynapse and post synapse” (GO:0099560) (Fig. 1*F* and supplemental Table S3). This suggests that the CSF reflects physiological or pathological proteomic alterations in the central nervous system, confirming the absence of contaminations that could affect the integrity of the obtained results.

### CSF Profiling of Canonical AD Patients Identifies Disease-Associated Proteins

**Fig. 2:**
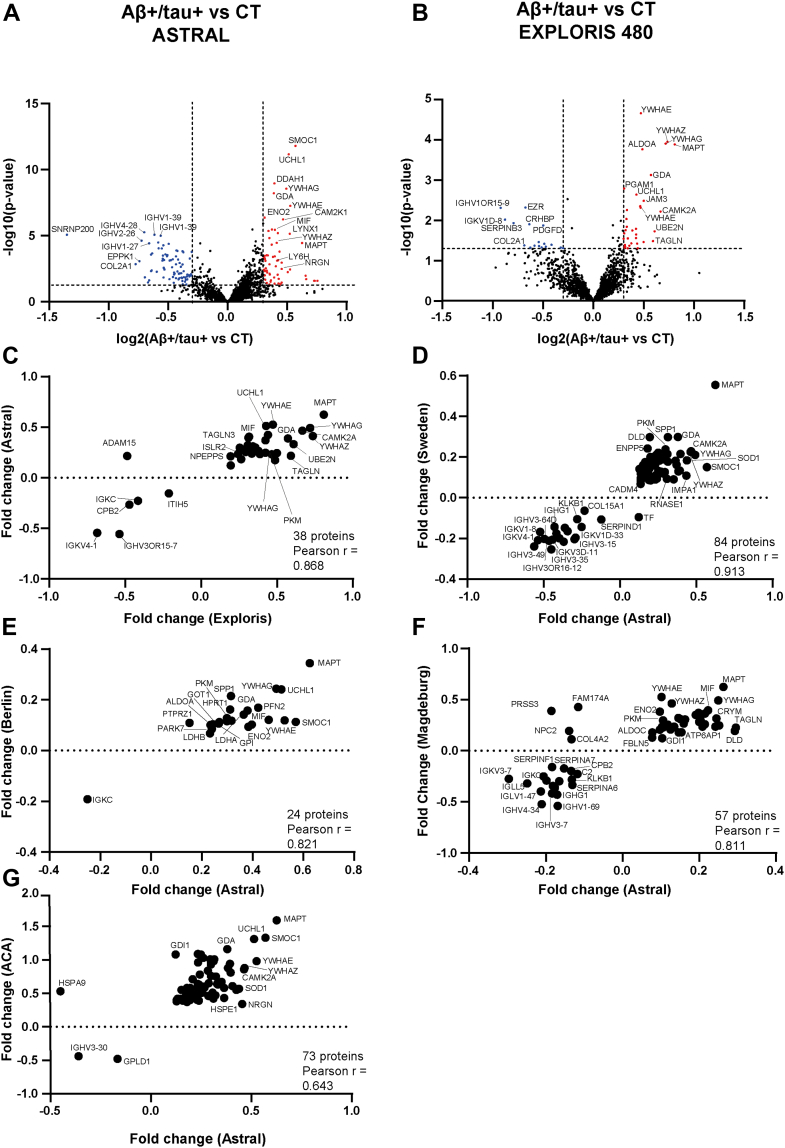
**Proteomic analysis of changes in the CSF of Aβ+/tau + AD patients**. *A* and *B*, analysis of protein changes between the Aβ+/tau + group and non-AD controls (CT), measured by an Astral (*A*) or Exploris 480 (*B*) mass spectrometer. Volcano plot showing the -log10 of *p*-values versus the log2 of protein ratio between Aβ+/tau + group and the non-AD control group. Proteins were arbitrarily considered as altered when their fold change was above 1.5 and *p*-value less than 0.05 (displayed by the *dotted lines* in the plot). Proteins more abundant in the CSF of AD patients are displayed in *red* and reduced proteins in *blue*. *C*–*G*, correlation for pairs of combinations between the protein Aβ+/tau + AD *versus* non-AD fold changes of our cohort analyzed by Astral and the validation cohort analyzed by Exploris 480 (*C*), our cohort analyzed by Astral and the Sweden cohort (*D*), the Berlin cohort (*E*) and the Madgeburg/Kiel cohort (*F*). Proteins shown in the plots differed significantly (*p* < 0.05) and consistently in each pair of cohorts. Data for the Sweden, Berlin, and Madgeburg/Kiel were extracted from (17). AD, Alzheimer’s disease; CSF, cerebrospinal fluid.

After validating the depth and completeness of CSF proteome profiling, as well as the quality of CSF samples, we next aimed to identify protein classifiers of AD through a quantitative analysis of CSF proteins. We initially compared samples from non-AD controls to those from the Aβ+/tau + AD patients. A total of 1584 proteins were identified in both groups. Among these, 61 proteins were significantly more abundant in the CSF of AD patients, including tau (displayed by the gene name MAPT) in agreement with ELISA results and consistent with the clinical classification of patients (Fig. 2*A* and supplemental Table S4). In addition to tau, the increased proteins included three members of the 14-3-3 family (YWHAE, YWHAZ, and YWHAG), along with other proteins of potential relevance to AD such as UCHL1, SMOC1, and macrophage migration inhibitory factor (MIF). A GO analysis revealed enrichment in biological processes related to “presynaptic endocytosis” (GO:0140238) and cellular components related to “presynaptic and postsynaptic cytosol” (GO:0099524; GO:0099523) (supplemental Table S3). Conversely, 78 proteins were significantly reduced in the CSF of the Aβ+/tau + AD patients compared to controls, including several immunoglobulins, SNRNP200, COL2A1, EPPK1, and others (Fig. 2*A* and supplemental Table S4). GO analysis of these reduced proteins did not indicate significant enrichment for any specific functional categories.

### Cross-Cohort Validation Confirms Robustness and Reproducibility of CSF Proteomic Signatures in AD

To validate the findings from our primary cohort, we compared our results with multiple independent datasets derived from different patient cohorts, analytical workflows, and mass spectrometry platforms. First, we retrospectively analyzed an internally generated dataset from a separate cohort of 24 AD patients and 18 non-AD controls. This cohort was processed using the same workflow, but peptides were analyzed on an Exploris 480 mass spectrometer. A total of 1492 proteins were identified in the CSF of both Aβ+/tau + AD patients and controls. Of these, 44 proteins were more abundant in AD patients, including tau and several 14-3-3 family members (YWHAE, YWHAZ, YWHAG, and YWHAH), consistent with our main dataset (Fig. 2*B* and supplemental Table S4). Nineteen proteins were reduced in the CSF of the Aβ+/tau + AD patients, including several immunoglobulins, EZR and COL2A1. Differentially abundant proteins of the two datasets, one generated with Astral on a larger cohort and the other with Exploris on a smaller cohort, were strongly correlated with a Pearson’s r = 0.868 (Fig. 2*C*).

Next, we compared our results with those from the reference study by Bader *et al*., which used a similar proteomic pipeline to profile CSF from Aβ+/tau + AD patients across three cohorts (Sweden, Magdeburg/Kiel and Berlin) using a Q Exactive HF-X Orbitrap (17). Our dataset showed high correlation with all three: r = 0.91 (Sweden), r = 0.81 (Magdeburg/Kiel), and r = 0.82 (Berlin) (Fig. 2, *D*–*F*). Most of the proteins identified as enriched in Aβ+/tau + AD patients in the Bader study, such as MAPT, YWHAG, YWHAZ, YWHAE, SMOC1, and MIF were also detected in our data.

**Supplementary Figure 3:**
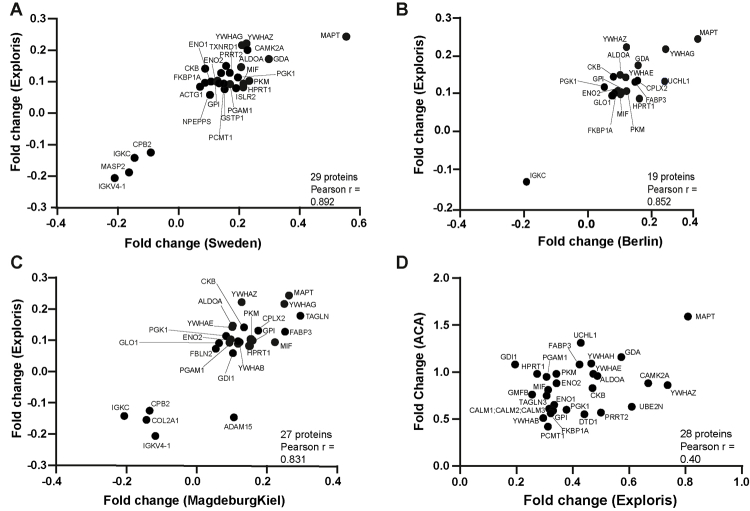
Correlation for pairs of combinations between the protein Aβ+/tau+ AD *versus* non-AD fold changes of our cohort analysed by Exploris and the Sweden cohort (D), the Berlin cohort (E), the Madgeburg/Kiel cohort (F) and the ACA cohort. Proteins shown in the plots differed significantly (*p* < 0.05) and consistently in each pair of cohorts. Data for the Sweden, Berlin and Madgeburg/Kiel were extracted from (17), and for the ACA cohort from the AD Workbench.

We further assessed concordance with a dataset from the ACA, where CSF samples from Aβ+/tau + AD patients and non-AD controls were analyzed using tandem mass tag labeling, Exploris 480 with “field asymmetric ion mobility spectrometry, and DDA (14). Despite methodological differences, a positive correlation was observed (Pearson’s r = 0.643; Fig. 2*G*), with proteins such as MAPT, YWHAZ, YWHAE, and SMOC1 being more abundant in the Aβ+/tau + AD patients of both datasets. Notably, our smaller dataset generated with the Exploris 480 also correlated well with the Bader *et al*. cohorts (r = 0.892, 0.831, and 0.852 for Sweden, Magdeburg/Kiel and Berlin, respectively) and showed moderate correlation with the ACA dataset (r = 0.40; supplemental Fig. S3).

Altogether, these analyses show a strong agreement between our data and previously published datasets, regardless of cohort size, mass spectrometer or proteomic method. This confirms the reproducibility and robustness of our workflow and supports the generalizability of CSF protein alterations in AD across different populations and platforms. These consistent findings reinforce the potential of the identified proteins as reliable biomarkers for AD.

### CSF Profiling of AD Patients with Normal tau Levels Reveals a Distinct Proteomic Pattern

**Fig. 3:**
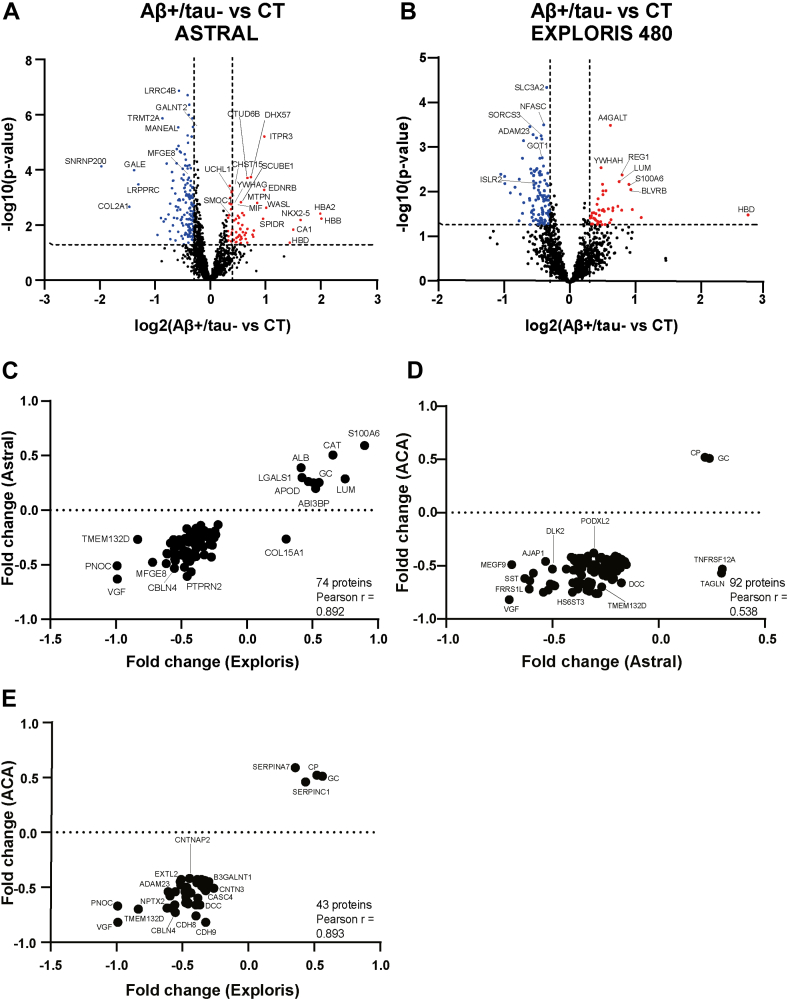
**Proteomic profiling of CSF from Aβ+/tau- AD patients**. *A* and *B*, analysis of protein changes between the Aβ+/tau-group and non-AD controls (CT), measured by an Astral (*A*) or Exploris 480 (*B*) mass spectrometer. Volcano plot showing the -log10 of *p*-values versus the log2 of protein ratio between the Aβ+/tau-group and the non-AD control group (CT). The threshold for statistical significance of protein changes was arbitrarily set at fold change above 1.5 and *p*-value less then 0.05 (displayed as *dotted curves* in the plot). Proteins more abundant in the Aβ+/tau-group are displayed as *red dots*, and proteins reduced displayed as *blue dots*. Selected proteins with a -log10 above 2 are named. *C*-*E*, correlation for pairs of combinations between the protein Aβ+/tau- AD *versus* non-AD fold changes of our cohort analyzed by Astral and the validation cohort analyzed by Exploris 480 (*C*), our cohort analyzed by Astral and the ACA cohort (*D*), and our cohort analyzed by Exploris 480 and the ACA cohort (*E*). Data for the ACA cohort were extracted from the AD Workbench, a publicly available dataset generated within the Alzheimer’s Disease Data Initiative. ACA, Alzheimer Center Amsterdam; AD, Alzheimer’s disease; CSF, cerebrospinal fluid.

Next, we profiled the CSF proteome of patients characterized by reduced CSF levels of Aβ_42_ but normal tau levels (Aβ+/tau-subgroup). To identify proteins associated with this subgroup, we compared the CSF of the Aβ+/tau-group with that of non-AD controls. A total of 1584 proteins were quantified in both groups (Fig. 3*A*). Sixty proteins were significantly more abundant in the Aβ+/tau-group, including carbonic anhydrase 1 (CA1), actin nucleation-promoting factor (WASL), and inositol 1,4,5-trisphosphate receptor type 3 (ITPR3). Conversely, 147 proteins were reduced in the CSF of Aβ+/tau- AD patients, including leucine-rich PPR motif-containing protein (LRPPRC), UDP-glucose 4-epimerase (GALE), collagen alpha-1(II) chain (COL2A1), and U5 small nuclear ribonucleoprotein 200 kDa helicase (SNRNP200). Although more abundant proteins were not significantly enriched for specific GO terms, the reduced were mainly proteins of the “synaptic cleft” (GO:0043083) and linked to “regulation of postsynaptic neurotransmitter receptor activity” (GO:0098962) (supplemental Table S2).

To validate these findings, we retrospectively analyzed an independent dataset generated from a smaller cohort of Aβ+/tau-patients, whose CSF samples had been profiled using an Exploris 480 mass spectrometer. A total of 1492 proteins were quantified (Fig. 3*B* and supplemental Table S4), and the observed proteomic changes correlated with those from our main dataset (Pearson’s r = 0.892) (Fig. 3*C*). Moreover, significant changes in our dataset also correlated with that generated from the ACA cohort (r = 0.538) (Fig. 3*D*) (14). Notably a direct comparison with the Bader *et al*. study was not feasible, as their dataset did not include Aβ+/tau– patients. However, when the dataset from the ACA was compared with our Aβ+/tau– versus control dataset from the smaller cohort, a strong correlation was observed (r = 0.893) (Fig. 3*E*), further supporting the consistency of the proteomic changes identified in this AD subgroup.

### Distinct CSF Proteomic Signatures Differentiate Tau-Positive and Tau-Negative AD Subtypes

**Fig. 4:**
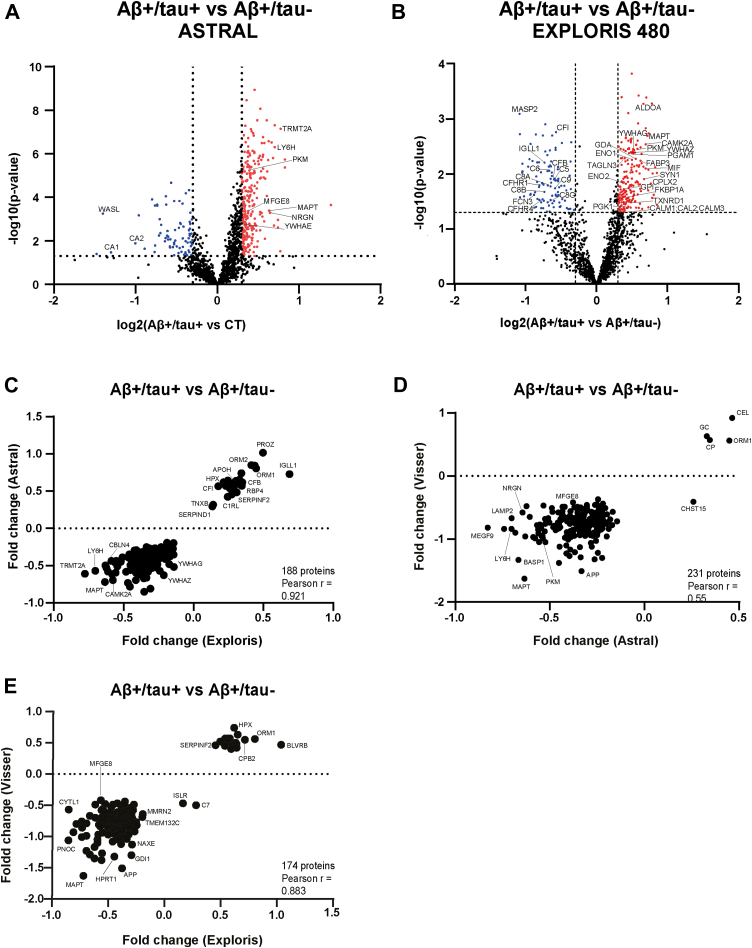
**Proteomic profiling of CSF from Aβ+/tau- AD patients**. *A* and *B*, analysis of protein changes between the Aβ+/tau + group and the Aβ+/tau-group, measured by an Astral (*A*) or Exploris 480 (*B*) mass spectrometer. Volcano plot showing the -log10 of *p*-values versus the log2 of protein ratio between the Aβ+/tau + group and the Aβ+/tau-group of AD patients. The threshold for statistical significance of protein changes was arbitrarily set at fold change above 1.5 and *p*-value less then 0.05 (displayed as *dotted curves* in the plot). Proteins more abundant in the Aβ+/tau + group are displayed as *red dots*. Proteins significantly reduced in the Aβ+/tau + group are displayed as *blue dots* (selected proteins are highlighted). *C*–*E*, correlation for pairs of combinations between the protein Aβ+/tau + AD versus Aβ+/tau- AD fold changes of our cohort analyzed by Astral, and the validation cohort analyzed by Exploris 480 (*C*), our cohort analyzed by Astral and the ACA cohort (*D*) and our cohort analyzed by Exploris 480 and the ACA cohort (*E*). Data for the ACA cohort were extracted from the AD Workbench, a publicly available dataset generated within the Alzheimer’s Disease Data Initiative. ACA, Alzheimer Center Amsterdam; AD, Alzheimer’s disease; CSF, cerebrospinal fluid.

To uncover molecular features distinguishing Aβ+/tau– from Aβ+/tau + AD patients, we directly compared their CSF proteomes. Of the 1584 proteins identified across both groups, 380 were differentially abundant (Fig. 4*A* and supplemental Table S4). Among these, 258 proteins were significantly increased in the CSF of Aβ+/tau + patients, including tau (MAPT) and YWHAE, proteins previously identified as markers of typical AD pathology. Their enrichment supports the notion that they reflect tau-driven neurodegeneration and are likely involved in tau aggregation and neurofibrillary tangle formation (30, 31, 32). GO analysis of proteins enriched in the Aβ+/tau + group revealed associations with regulation of postsynaptic neurotransmitter receptor activity (GO:0098962) and synapse adhesion between presynapse and postsynapse (GO:0099560), underscoring the neurodegenerative processes underlying classical AD (supplemental Table S3). In contrast, 72 proteins were more abundant in the Aβ+/tau-group. These included the carbonic anhydrase 1 and 2 (CA1, CA2) and actin nucleation-promoting factor (WASL). GO analysis did not associate these proteins with specific biological processes or cell components. As with previous comparisons, we retrospectively validated our findings using the dataset generated from a second cohort of AD patients and analyzed with an Exploris 480 mass spectrometer. In this smaller cohort, the comparison between Aβ+/tau– and Aβ+/tau + patients revealed 283 proteins significantly altered between the two groups: 182 were more abundant in the Aβ+/tau + group—including tau—and 101 were more abundant in the Aβ+/tau– group (Fig. 4*B* and supplemental Table S4). The differential proteomic profile generated by Astral showed strong agreement with that obtained from the smaller cohort analyzed by Exploris, with a Pearson correlation coefficient of r = 0.92 (Fig. 4*C*). A moderate correlation was also observed between the Astral dataset and that from ACA (r = 0.55) (Fig. 4*D*), while the Exploris dataset from the smaller cohort correlated well with the ACA dataset (r = 0.88) (Fig. 4*E*).

Overall, these findings reinforce that, despite similar clinical presentations, Aβ+/tau- and Aβ+/tau + AD patients display distinct CSF proteomic profiles, both from each other and from non-AD controls, suggesting that they may represent biologically distinct subtypes of Alzheimer’s disease.

### Machine Learning Uncovers Distinct Proteomic Signatures of Aβ+/tau+ and Aβ+/tau– Alzheimer's Disease Subtypes

**Fig. 5:**
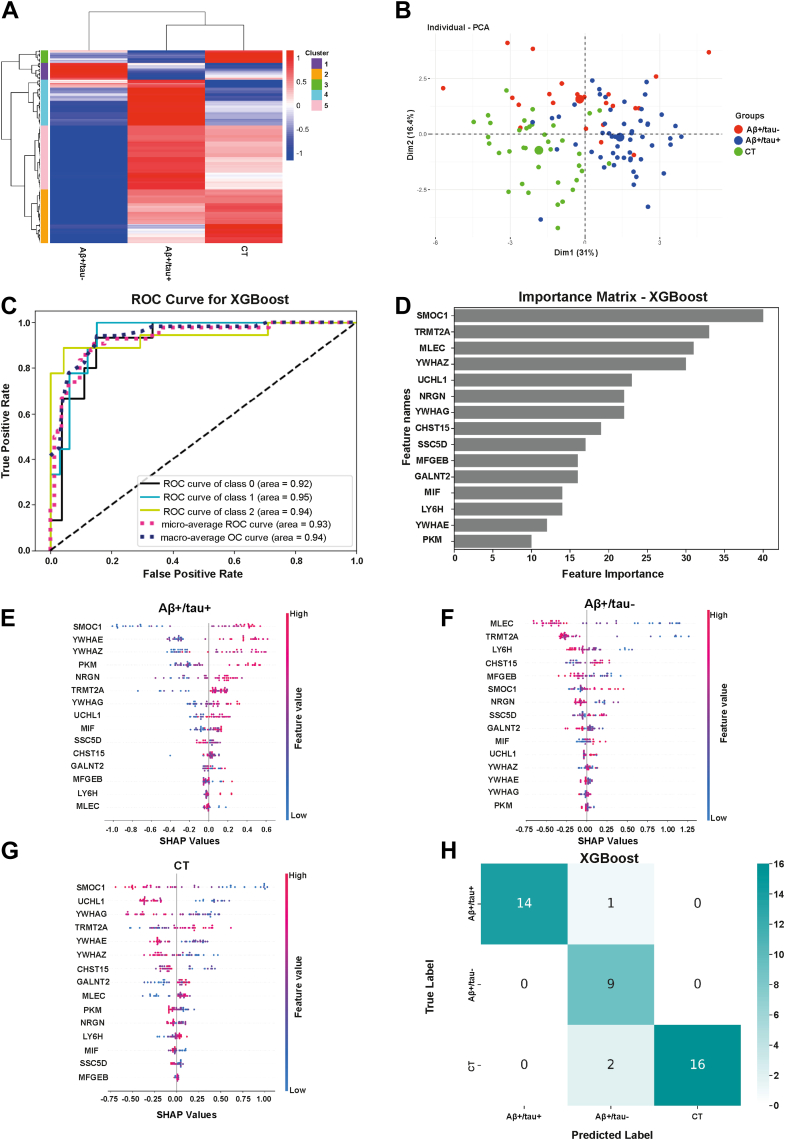
**Proteomics and machine learning identify 15 protein classifiers that discriminate Aβ+/tau+, Aβ+/tau−, and non-AD patients**. *A*, heatmap showing hierarchical clustering of 342 differentially abundant proteins across the three groups, revealing five distinct clusters based on abundance patterns. *B*, principal component analysis (PCA) of Aβ+/tau+, Aβ+/tau-, and non-AD controls based on 352 proteins differently abundant across the three groups. *C*, ROC curves from the XGBoost model trained on the 15 selected protein classifiers. *D*, feature importance plot from the XGBoost model identifying the *top* 15 proteins contributing to classification accuracy. *E*–*G*, SHAP (Shapley additive explanations) summary plots showing the individual contribution and distribution of the top 15 protein features to the prediction of Aβ+/tau+ (*E*), Aβ+/tau- (*F*), and control (*G*) groups. *H*, confusion matrix showing prediction accuracy of the XGBoost model on the test set (30% of data). AD, Alzheimer’s disease; ROC, receiver operating characteristic.

Recent advances in machine learning have enabled to uncover biomarkers that may not be apparent through conventional statistical methods (33). Thus, we used machine learning to identify the minimal number of protein classifiers that most accurately distinguish Aβ+/tau+, Aβ+/tau-, and non-AD individuals. First, we applied multisample testing with FDR correction (q < 0.05) to our dataset. This revealed 342 differentially abundant proteins. After Z-scoring, hierarchical clustering based on these proteins achieved clear separation among the three patient groups (Fig. 5*A*). These proteins formed five major clusters: (1) proteins more abundant in Aβ+/tau- AD than in the other two groups (e.g. SSC5D and CHST15); (2) proteins with lower abundance in the Aβ+/tau- AD than in the other two groups, (e.g. MGFEB and GALNT2); (3) proteins with higher abundance in non-AD controls; (4) proteins enriched in Aβ+/tau + AD, such as tau (MAPT) and the 14-3-3 proteins (e.g. YWHAE, YWHAZ, and YWHAZ); and (5) proteins reduced in Aβ+/tau- AD but elevated in controls (e.g. MLEC and LY6H). Principal component analysis of the same 342 proteins further supported the stratification of individuals into the three diagnostic groups, showing a separation between Aβ+/tau+, Aβ+/tau−, and non-AD cases (Fig. 5*B*).

**Supplementary Figure 4:**
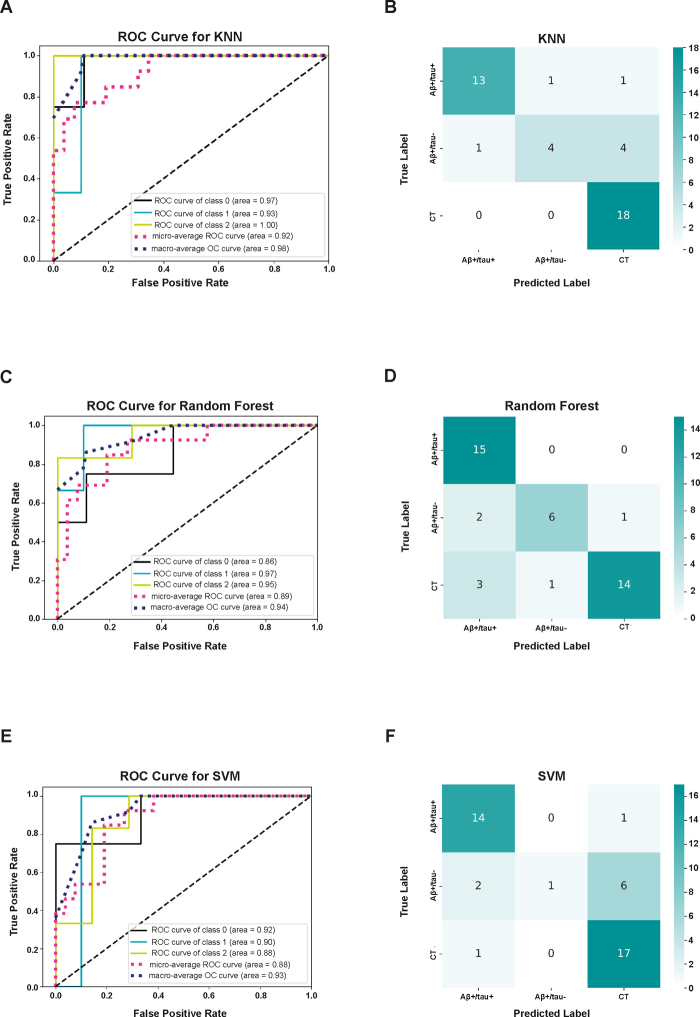
ROC curves and confusion matrix showing the performance of a KNN model (*A* and *B*), a Random Forest model (*C* and *D*) and a SVM model (*E* and *F*) to classify Aβ+/tau+, Aβ+/tau− and non-AD controls (CT), tested on 30% of our dataset generated by Astral.

**Supplementary Figure 5:**
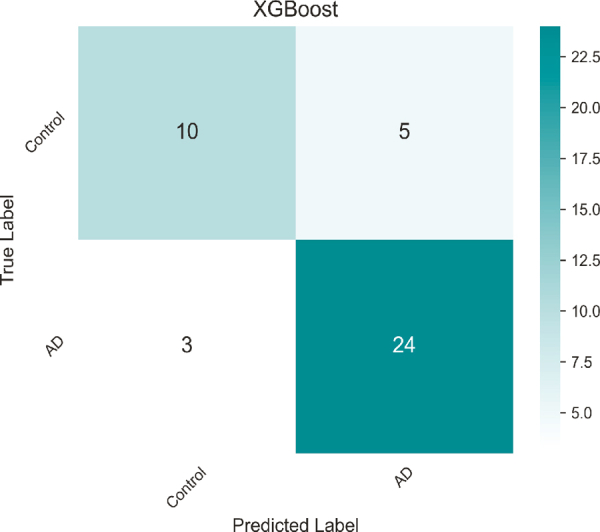
ROC curves and confusion matrix showing the performance of a KNN model (*A* and *B*), a Random Forest model (*C* and *D*) and a XGBoost model (*E* and *F*) to classify AD patients and non-AD controls (CT), without preliminary stratification in Aβ+/tau+, Aβ+/tau-, tested on 30% of our dataset generated by Astral.

We next evaluated the performance of four machine learning algorithms—XGBoost, SVM, KNN and Random Forest—in classifying Aβ+/tau- AD, Aβ+/tau + AD and non-AD control patients. To this end, 70% of the dataset was used for training. Among the tested models, the gradient boosting decision tree algorithm (XGBoost) showed the highest performance, achieving an area under the curve (AUC) of 0.93, followed by KNN and Random Forest (both AUC = 0.83), and SVM (AUC = 0.76) (Fig. 5*C* and supplemental Fig. S4). XGBoost identified a subset of 15 proteins as key discriminatory features (including SMOC1, TRMT2, MLEC, YWHAZ, UCHL1, NRGN, YWHAG, CHST15, SSC5D, MFGE8, GALNT2, MIF, LY6H, YWHAE, and PKM), while the remaining differentially regulated proteins among the 342 initially identified had negligible contribution to the classification (Fig. 5*D*). SHAP analysis further revealed that SMOC1 was the top-ranked feature driving separation between the Aβ+/tau+ and non-AD controls, while MLEC was the most important in classifying Aβ+/tau- AD patients (Fig. 5, *E*–*G*). On the test set (30% of the data), the XGBoost model correctly classified 39 out of 42 patients (Fig. 5*H*). Importantly, when the same model was applied to classify AD versus non-AD patients without stratifying by tau status, its performance dropped significantly and misclassified 8 out of 42 patients (supplemental Fig. S5) indicating that while machine learning can effectively discriminate AD patients from non-AD controls, its full potential is realized when biologically relevant subgroups, such as Aβ+/tau+ and Aβ+/tau- AD, are considered in the classification strategy.

**Fig. 6:**
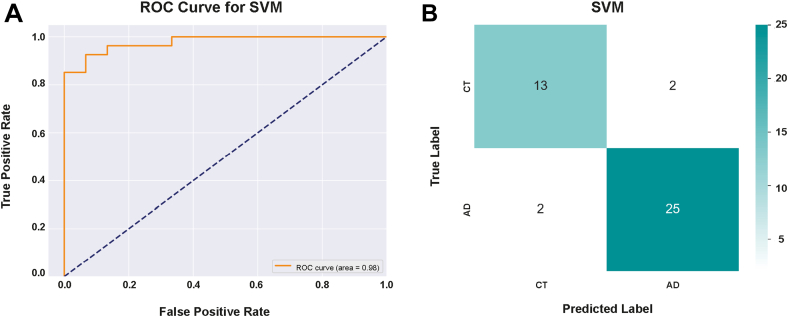
**Machine learning enables accurate classification of AD patients versus non-AD controls**. *A* and *B*, ROC curve and confusion matrix showing the classification performance of a SVM model trained on six selected protein classifiers of AD patients or non-AD controls (CT). AD, Alzheimer’s disease; ROC, receiver operating characteristic; SVM, support vector machine.

**Supplementary Figure 6:**
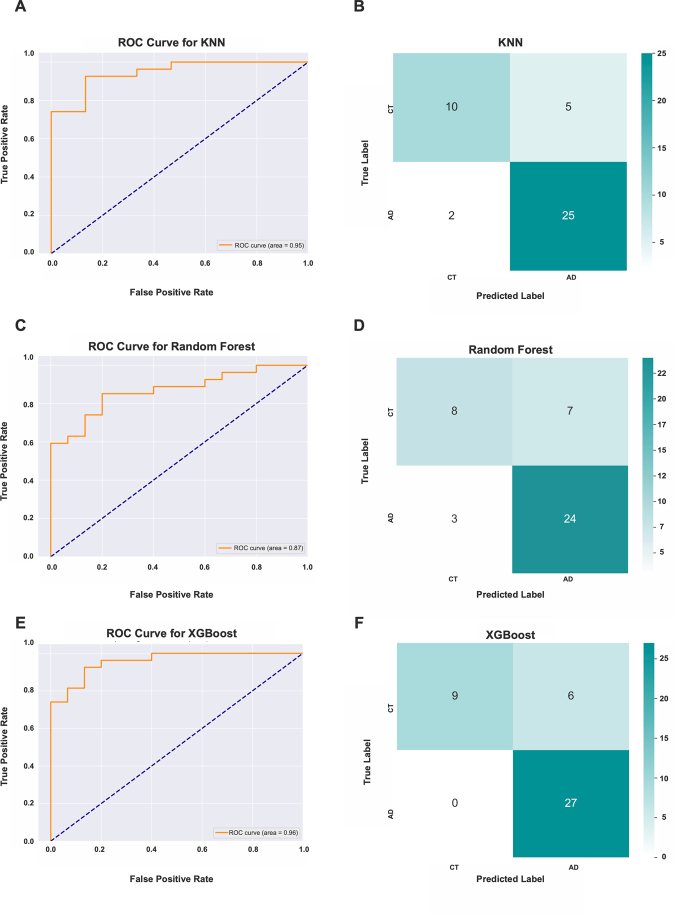
ROC curves and confusion matrix showing the performance of a SVM model (*A* and *B*), a KNN model (*C* and *D*) and a Random Forest model (*E* and *F*) to classify Aβ+/tau+, Aβ+/tau− and non-AD controls (CT), tested on the ACA dataset.

Next, we trained a model to classify AD patients versus non-AD controls, without prior stratification into Aβ+/tau- and Aβ+/tau + subgroups. From the dataset, 55 proteins were found to be differentially abundant between the two groups and were used as input for machine learning algorithms, as previously described. Among them, the SVM algorithm achieved the highest classification accuracy, correctly identifying 38 out of 42 patients (Fig. 6, *A* and *B*). Only six proteins substantially contributed to the predictive power of the SVM model (DDAH1, SMOC1, YWHAG, UCHL1, MAN2A1, and YWHAZ). XGBoost followed with 36 correct classifications, while KNN and Random Forest classified 34 and 32 patients correctly, respectively (supplemental Fig. S6). These findings underscore the versatility of our proteomics and machine learning workflow, which can be effectively applied to both general AD classification and more biologically defined patient subgroups.

### In Silico Validation of Subtype-Specific AD Classifiers Using an Independent Cohort

**Fig. 7:**
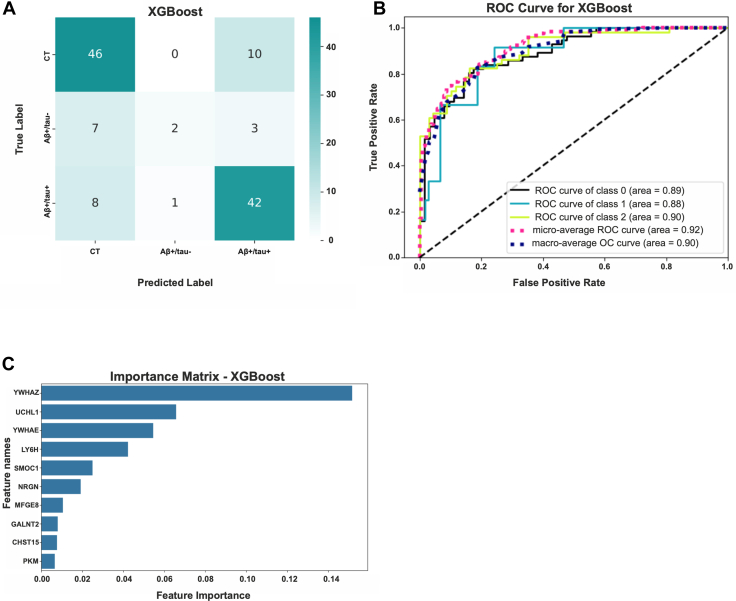
**The trained model accurately classifies Aβ+/tau+, Aβ+/tau−, and non-AD patients of the ACA cohort**. *A* and *B*, ROC curve and confusion matrix showing the performance of the XGBoost model based on the 15 protein classifiers tested on 118 patients, including Aβ+/tau+, Aβ+/tau−, and non-AD controls (CT), from the ACA cohort. *C*, feature importance plot from the XGBoost model identifying the proteins most contributing to classification accuracy when applied to the ACA cohort. ACA, Alzheimer Center Amsterdam; AD, Alzheimer’s disease; ROC, receiver operating characteristic.

To evaluate the broader applicability of our model in diagnosing Aβ+/tau+ and Aβ+/tau-subtypes of AD, we tested its performance on an independent cohort of AD patients from the ACA. We applied our previously trained XGBoost model, based on 15 classifier proteins, to the ACA CSF proteomic dataset. Fourteen of these proteins were available in this dataset (TRMT2 was not detected and therefore excluded). Despite this omission, the model achieved a good classification accuracy (Fig. 7, *A* and *B*). Notably, when applied to the ACA dataset, the protein mostly contributing to the discriminative power of the model was YWHAZ (Fig. 7*C*). When the same 14 proteins were used as input features in other machine learning algorithms, classification remained robust, with AUC values of 0.90 for SVM, 0.85 for Random Forest, and 0.83 for KNN (supplemental Fig. S6).

**Fig. 8:**
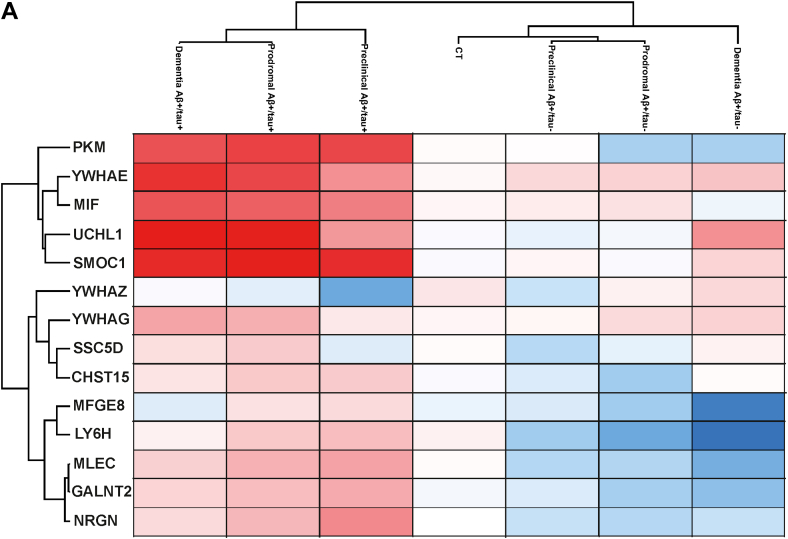
**Protein classifiers of AD can be found in the CSF before the onset of symptoms**. *A*, heatmap showing hierarchical clustering of Aβ+/tau+, Aβ+/tau- and non-AD controls (CT), further stratified in patients before symptoms appear (preclinical), patients with mild cognitive impairment (prodromal) and patients with dementia (clinical), based on the 15 protein classifiers and applied to the dataset extracted from the AD Workbench, a publicly available dataset generated within the Alzheimer’s Disease Data Initiative. AD, Alzheimer’s disease; CSF, cerebrospinal fluid.

The early detection of CSF biomarkers for diagnosing and subclassifying AD is critical, as it improves diagnostic accuracy during prodromal stages and may support the development of personalized therapeutic strategies. To assess whether our 14 classifier proteins could serve this purpose, we analyzed their levels across different clinical stages of AD using the ACA dataset (34), which included 107 individuals in the preclinical stage (asymptomatic), 103 with MCI (prodromal), and 209 with dementia (clinical stage) (Fig. 8). Four of these classifier proteins (YWHAE, MIF, UCHL1, and SMOC1) were elevated in the CSF of Aβ+/tau + AD patients even before the onset of symptoms and remained consistently high throughout disease progression. In Aβ+/tau-patients, these proteins also increased over time, though to a lesser extent. Conversely, PKM was elevated at all stages in Aβ+/tau + patients but declined as the disease progressed in Aβ+/tau-individuals. YWHAG, SSC5D, and CHST15 showed early increases in Aβ+/tau + patients, while in Aβ+/tau-patients their upregulation occurred at later stages. Notably, YWHAQ levels were consistently higher in Aβ+/tau-patients compared to Aβ+/tau + patients across all stages. Finally, MFGE8, LY6H, MLEC, GALNT2, and NRGN were elevated in Aβ+/tau + patients relative to controls at all stages, whereas in Aβ+/tau-patients these proteins were reduced.

In conclusion, these findings establish a robust and high-resolution proteomic workflow for CSF analysis that not only confirms established biomarkers of canonical AD but also reveals distinct and reproducible molecular signatures that appear at early stages of the disease and well distinguish Aβ+/tau+ and Aβ+/tau-subtypes, paving the way for improved biomarker-based stratification of AD.

## Discussion

AD is characterized by progressive cognitive decline that impairs daily functioning and quality of life. Its underlying pathology, including Aβ plaque accumulation and neurofibrillary tangle formation, begins years before clinical symptoms emerge (35). In recent years, it has become clear that AD is a multifactorial disorder, with distinct patient subgroups characterized by different pathophysiological mechanisms beyond Aβ accumulation and tau pathology. This complexity underscores the urgent need for biomarkers that can enable early diagnosis and accurate patient stratification to support personalized medicine and more effective clinical trial design (3, 4).

In this study, we extended a recently developed pipeline based on DIA for comprehensive identification and quantification of CSF proteins with high-resolution analysis using an Astral mass spectrometer. This platform enabled an unprecedented, in-depth characterization of the CSF proteome of AD patients. Our analysis identified changes in the CSF of canonical AD patients, hence characterized by decrease in CSF levels of Aβ42 and an increase of tau, that were consistent with observations from several independent studies on different cohorts and using various proteomic pipelines and mass spectrometer platforms (14, 17). We found that in addition to tau (MAPT), several 14-3-3 proteins (YWHAE, YWHAG, and YWHAZ) were augmented in the CSF of AD patients. The increase of these proteins in the CSF of AD patients reflects tau pathology. In AD, tau protein becomes abnormally phosphorylated, detaches from microtubules, and forms toxic aggregates known as neurofibrillary tangles. This process causes neuronal damage, leading to the release of tau into the CSF, along with 14-3-3 proteins that interact with tau (36, 37). Beyond tau and 14-3-3 proteins, we also identified elevated levels of SMOC1, MIF, NRG1, and UCHL1 in the CSF of AD patients—findings that were corroborated by other proteomic and immunoassay-based studies, including those using Olink (8, 38). SMOC1, a secreted extracellular matrix protein expressed by reactive astrocytes, is among the earliest altered proteins in AD. It has been shown to interact with Aβ and modulate disease progression (39). MIF, a proinflammatory cytokine, was also consistently elevated. MIF is known to drive glial activation, promote tau hyperphosphorylation, and contribute to oxidative stress and disease progression in AD (40). Similarly, NRG1 (neuregulin-1) was upregulated in our dataset and others, supporting its role in synaptic dysfunction, cognitive decline, and microglial dysregulation in 10.13039/100020014AD (8, 14, 41, 42). The reproducible detection of these protein changes across independent cohorts of AD patients, analytical pipelines, and platforms highlights their robustness as candidate CSF biomarkers that may improve diagnostic precision and complement the ATN classification system by capturing additional pathophysiological processes in AD.

Next, we analyzed changes in the CSF proteome of a subgroup of AD patients characterized by reduced Aβ42 levels but normal tau levels. This less-characterized form of 10.13039/100020014AD underscores the need for additional biomarkers beyond tau to support early diagnosis. Our analysis revealed that the CSF proteome in this group differed significantly not only from non-AD controls, as expected, but also from typical Aβ+/tau + AD patients. These findings are consistent with a previous study on a larger ACA cohort (14). We found that proteins such as GALNT2, TRMT2A, and MFGE8 were reduced in the CSF of AD patients belonging to this subgroup. Unlike GALNT2 and TRMT2A, whose evidence of involvement in AD are limited, MFGE8 (a.k.a. medin) is strongly linked to AD, as it interacts directly with amyloid-β to promote its aggregation and it is associated with cognitive decline (43). Given its role in amyloid pathology, MFGE8 is under investigation as a therapeutic target for preventing Aβ-driven neurodegeneration. Its reduction in this AD subgroup suggests that such therapeutic strategies may not be effective for these patients. Overall, these findings reinforce the value of CSF proteomic profiling in identifying biomarkers that aid in early diagnosis and patient stratification across distinct AD subtypes.

After deeply profiling the CSF proteome of AD patients, we applied machine learning to identify protein classifiers capable of distinguishing Aβ+/tau+, Aβ+/tau-, and non-AD patients. This approach led to the identification of a panel of 15 protein classifiers. We used these proteins to train a gradient-boosting model that effectively distinguished AD patients with high versus normal tau levels. To test its robustness, we validated the model using the independent ACA cohort, where it maintained strong discriminatory power across the three groups. Given that tau is currently the most reliable predictive, prognostic, and diagnostic CSF marker—rising early in AD and progressing with disease severity—we further assessed whether our classifier proteins might complement tau (34, 44). Exploiting the ACA dataset, which includes patients across preclinical, prodromal, and clinical stages of AD, we examined how our 15-protein signature evolved with disease progression. Notably, several of these proteins, including YWHAE, MIF, UCHL1, and SMOC1, were elevated at early disease stages and continued to increase over time, supporting their potential as early biomarkers (45, 46). In the Aβ+/tau-patients, where diagnosis currently relies solely on amyloid-β measurements, additional biomarkers could enhance the diagnostic accuracy. Notably, proteins such as MFGE8, LY6H, MLEC, GALNT2, and NRGN, were consistently reduced in this subgroup, even before symptom onset, underscoring their potential as early biomarkers for this distinct form of AD. These findings may also guide personalized treatment strategies, particularly as tau-targeted immunotherapies have shown efficacy in Aβ+/tau + patients, as demonstrated in a phase 2 clinical trial (40). Integrating our 15 protein with conventional systems for AD diagnosis, such as the ATN framework, could significantly improve diagnostic precision, enable better patient stratification, inform tailored therapies, optimize clinical trial design, and ultimately enhance treatment outcomes.

In conclusion, recent advances in mass spectrometry have enabled deeper insights into the CSF proteome and its changes in AD. Using a high-resolution proteomic pipeline, we achieved extensive CSF coverage and identified a 15-protein signature that distinguishes Aβ+/tau+, Aβ+/tau- and non-AD patients. Consistent with findings from other cohorts and platforms, this signature may complement the ATN framework to improve AD diagnosis and stratification.

## Data Availability

The mass spectrometry proteomics data have been deposited to the ProteomeXchange Consortium via the PRIDE partner repository with the dataset identifier PXD063058.

## Supplemental Data

This article contains supplemental data.

## Conflict of Interest

The authors declare no competing interests.
